# Butterfly Encryption Scheme for Resource-Constrained Wireless Networks †

**DOI:** 10.3390/s150923145

**Published:** 2015-09-15

**Authors:** Raghav V. Sampangi, Srinivas Sampalli

**Affiliations:** Faculty of Computer Science, Dalhousie University, 6050 University Ave., PO Box 15000, Halifax, NS B3H 4R2, Canada; E-Mail: srini@cs.dal.ca

**Keywords:** security, key management, authentication, resource-constrained wireless networks, RFID, Wireless Body Area Networks

## Abstract

Resource-constrained wireless networks are emerging networks such as Radio Frequency Identification (RFID) and Wireless Body Area Networks (WBAN) that might have restrictions on the available resources and the computations that can be performed. These emerging technologies are increasing in popularity, particularly in defence, anti-counterfeiting, logistics and medical applications, and in consumer applications with growing popularity of the Internet of Things. With communication over wireless channels, it is essential to focus attention on securing data. In this paper, we present an encryption scheme called Butterfly encryption scheme. We first discuss a seed update mechanism for pseudorandom number generators (PRNG), and employ this technique to generate keys and authentication parameters for resource-constrained wireless networks. Our scheme is lightweight, as in it requires less resource when implemented and offers high security through increased unpredictability, owing to continuously changing parameters. Our work focuses on accomplishing high security through simplicity and reuse. We evaluate our encryption scheme using simulation, key similarity assessment, key sequence randomness assessment, protocol analysis and security analysis.

## 1. Introduction

The Internet of Things (IoT) paradigm, coupled with developments in related domains such as cloud computing, is creating a more connected world, with sensors, unique identification and artificial intelligence [1]. This has meant that technologies such as wireless body area networks (WBAN, which facilitate remote healthcare and assisted living) and radio frequency identification (RFID, facilitating unique identification of objects) are increasing in popularity, in an increasingly automated world, with intelligent devices.

RFID systems consist of RFID tags, RFID readers and backend servers. RFID tags are electronic circuits (with or without a power source) containing a unique identifier, which helps record and identify each object uniquely, among a set of other objects. RFID readers or interrogators are devices that initiate communication with these tags, and in case of passive tags without a power source, energize them sufficiently for them to be able to respond. RFID readers transmit the tag’s response to a backend server, querying it for further information about the object represented by the tag. On validating the tag, the server transmits the information to the reader, with optional information to the tag [2]. RFID tags, especially passive tags without power source, can use the reader’s signal strength to stay alive very briefly, during which time to perform any required computations and respond with the unique identifier. WBAN systems, on the other hand, contain on-body sensors and a hub (also a node on the body). The hub is usually connected to a personal server (PS), such as a cellular phone or a computer, which is further connected to the monitoring server at the hospital. The hub collects all health data recorded by individual sensors and transfers this to PS, which then forwards it to the monitoring server [3]. Because WBAN sensors are deployed on the human body and have varied length of deployment, optimizing resource usage between sensing and communication becomes critical. A common mechanism is to let the sensors enter “sleep” state, which can be woken up by the hub for communication [4]; very similar to RFID tags.

We have considered the examples of RFID tags and WBAN sensors here to illustrate the characteristics of some of the devices that are part of the IoT. Factors such as reduced size, required functionality, longevity in the deployed environment and low manufacturing cost in these technologies imply that these devices typically have resource restrictions.

With communication being mostly wireless in such application environments, data security assumes focus. However, the ability of a manufacturer to include sophisticated cryptography for data security is limited by the factors imposing resource restrictions. This calls for techniques that are optimized or lightweight, and which do not require considerable resources when deployed on these devices. Furthermore, with encryption algorithms typically published, the strength of the cryptosystems rests on the security of the key [5].

In our work, we present an encryption scheme based on pseudorandom number generators (PRNG, discussed in Section 3) for resource-constrained wireless networks. We refer to our scheme as the *Butterfly encryption scheme*. Our work employs a technique to update PRNG seeds with each message transmission that has been inspired by the Butterfly effect [6,7]. Our seed update mechanism (Section 3.1), in addition to parameters such as timestamps, ensures generation of a new key for each message encryption, making it also context-specific (owing to varied timestamp, messages and entities). Our scheme also includes a message signature generation mechanism, which is used to accomplish integrity verification and mutual authentication. The Butterfly encryption scheme was initially proposed for RFID systems [8]. We generalize it and extend it to WBANs in Section 4. To evaluate our work (Section 5), we consider key similarity and randomness assessment, followed by complexity assessment and security analysis. This combination of analyses enables us to understand the nature of the keys generated by our approach, and assess its complexity, in an attempt to verify its security and applicability to resource-constrained wireless networks.

## 2. Related Work

In resource-constrained devices, especially RFID tags and WBAN sensors, any improvement in efficiency in terms of reuse in circuits or functions would be beneficial. Typically, one popular choice for key generation in such systems are PRNG, since they generate unique sequences given different seeds, and depending on the algorithm, can operate with large periods without repeating sequences. PRNG algorithms such as Blum–Blum–Shub [9], J3Gen [10] and lightweight PRNG proposals by Martin *et al.* [11] are among many such algorithms proposed for cryptographic and resource-constrained applications. PRNG algorithms have become a very important component of cryptographic algorithms and protocols. In a typical setting, regular communication is usually preceded by a separate key agreement phase. Alternatively, key exchange and agreement can be combined with the regular message exchange to reduce overhead. Furthermore, separate functions (typically message digest generation algorithms [12]) are used to accomplish authentication, in a process that may involve the presence of a trusted certificate authority or a trusted enterprise server [13,14,15]. In this section, we discuss some of these approaches to key management and authentication in RFID and WBANs.

Often, mathematical hash algorithms are included as part of a symmetric cryptosystem for the purpose of facilitating message integrity verification. In some approaches, hash algorithms are also used to assist in authentication. One such approach is the application of the new SHA-3 standard (Keccak algorithm) [16] to compute message digests using pseudorandom numbers, keys and RFID tag IDs, proposed by Dong *et al.* [12]. The hash also contains an internally updated key, while the pseudorandom number is updated with each communication. In other approaches, hashes are used with digital signatures and trusted third party certificates for authentication. A different take on such mechanisms is to use a public key generator (PKG) to compute and distribute public keys and associated parameters [14]. The PKG computes and distributes partial public and secret keys to entities. When an entity needs to generate a signature, it uses its own identifier as a partial public key and requests the corresponding secret key from the PKG. Using the parameters generated by the PKG, the entity is able to generate its own certificateless signatures for authentication. This is a way of accomplishing authentication and non-repudiation by association with PKG, and implicitly a certificate authority. Their approach scheme does not specify whether it is designed to authenticate entities on each update. This is a factor to consider because of the increased load that public key functions and complex mathematical functions (such as exponentiation) will place on an already resource-constrained entity.

To exchange keys and accomplish authentication in WBANs and RFID systems, various mechanisms have been employed. An interesting approach to exchange keys in WBANs is suggested by Kovacevic *et al.* [15]. They use the visible light channel from a light source to establish secret keys and radio channel for authentication. The light source is generated using a multi-touch screen, using on-off keying modulation to transmit the keys to the body sensors, which need to be placed in such a way as to be able to recognize the light source. This key is used to generate a keyed hash that is used as one of the parameters for authentication. Their approach adds an additional burden on the WBAN user to place the sensors close to or on the light source for key exchanges, which is more of an inconvenience. This is in addition to the limitation of the keys being exchanged literally in the open via a light source. In RFID systems, Choi *et al.* [17] propose the use of physically unclonable functions (PUF), which are based on physical (electrical) characteristics. However, their work requires that challenge-response pairs are pre-computed using the PUF-based function and stored on the devices. It is to be noted that PUF-based implementations are subject to high sensitivity to changing physical characteristics of the tag.

While their work employs physical channel key exchange, Zhu *et al.*’s work [13] employs symmetric cryptography for key exchange and authentication. Their work includes a block-wise key update technique with XTEA encryption. Keys are updated block-wise (subkeys) and each block is transmitted by the server, encrypted using XTEA cipher. The subkeys are updated on successful authentication. With each update linked to the previous keys in this transmit-acknowledge mechanism of operation, this scheme is prone to de-synchronization attack. This can be accomplished by an attack blocking the key update message and transmitting an unrelated message in its place. This would cause the tag to roll back its update, causing de-synchronization.

Asymmetric cryptography, no matter how computationally extensive, is something that is being suggested for use in RFID and WBANs. The expectation (as per IEEE 802.15.6 standard for WBANs [4], and the EPCglobal [18] and ISO/IEC 29167-1:2014 specifications for RFID tags [19]) is for systems to employ asymmetric algorithms to accomplish several or all aspects of security. As a possible way to get the best of both symmetric and asymmetric techniques, Liu *et al.*’s work [20] suggests ways to use techniques based on ECDH (Elliptic curve-based Diffie-Hellman exchange) or AES (Advanced Encryption Standard) to accomplish key agreement and authentication among entities. However, as identified by the ISO/IEC 29167-1:2014 specification, use of sophisticated cryptography “impacts power consumption and processing time for the RFID components and may degrade system performance” [19], which would inevitably mandate increase in resources with requirements to perform exponentiation and Elliptic Curve Cryptography point multiplication for various computations, thereby increasing the cost.

Alternative approaches for security in resource-constrained networks also include using stream ciphers, such as the work by Luo *et al.* [21] and Shen *et al.* [22]. Luo *et al.*’s work employs the WG-7 stream cipher for RFID systems, while the work by Shen *et al.* uses PRNG and non-linear filters to compute hash values for authentication.

In our approach, we discuss a mechanism of continuously updating PRNG seeds that is naïvely based on the Butterfly effect [6,7]. We use this technique to update seeds in generating keys for encryption, which are updated with each message encryption and transmission. We also present an encryption scheme that employs both these techniques and discuss the protocol of operation. Our scheme is lightweight and facilitates reuse of common modules, which helps in reducing resource utilization in resource-constrained devices. Our approach also capitalizes on the deterministic nature of PRNG and on synchronized updates to the PRNG seeds to accomplish key generation, as well as mutual authentication.

## 3. Butterfly Encryption Scheme

*Butterfly effect* is a concept in chaos theory, defined by Poulin [7] (originally coined by Lorenz [6] as “hypersensitivity to perturbation”. This means that in a nonlinear deterministic system, if the initial conditions are changed ever so slightly, there will be drastic changes in the output of a later state. The Butterfly seed generation algorithm and the associated encryption scheme for RFID systems (A preliminary version of this work, intended only for RFID systems was presented at and has been published in the proceedings of the 22nd International Conference on Software, Telecommunications and Computer Networks (SoftCOM) 2014 [8].) are inspired by this concept. The basic principle behind the Butterfly seed generation algorithm is this—*changing a single bit in the seed of a pseudorandom number generator (PRNG) will change the output of the PRNG significantly*. In this section, we discuss briefly the seed update algorithm, which is a naïve adaptation of the concept of Butterfly effect, and we summarize the encryption scheme that employs it.

### 3.1. Butterfly Seed Generation Algorithm

PRNG are employed by cryptographic algorithms typically for generating encryption keys or nonces for other purposes. PRNG are inherently deterministic [23,24]. Their application is common in lightweight systems that have severe resource constraints that limit their ability to perform sophisticated computations for achieving security (e.g., lightweight passive RFID systems) [10,11,25,26]. Their deterministic nature, combined with a changing seed, can make the PRNG into a powerful authentication/simple key generation system, useful for RFID applications.

Let us consider a PRNG g() that generates *n*-bit pseudorandom numbers. Entities Alice and Bob, when synchronized with a particular initial seed, will be able to demand specific random numbers from each other. A simple authentication scheme could have Alice requiring that Bob respond with the $x^{th}$ random number generated using such a synchronized PRNG, to which Bob’s response could include its own demand that Alice respond with the ${\left(x+offset\right)}^{th}$ random number. To increase unpredictability, which would ultimately lead to increased security, we explored another way of updating the seeds that would also facilitate synchronization and increased uncertainty.

This uses the naïve adaptation of the Butterfly effect discussed earlier in this section. This approach uses state identifiers to (a) update the PRNG seed to a specific state; and (b) facilitate synchronization among entities. This scheme is thus able to facilitate entities to ‘generate’ encryption keys instead of exchanging keys and as a result generate parameters that enable mutual authentication.

Let us consider that the PRNG operation is governed by Equation (1)

(1) $$r=\{\;g\left(s_{j}\right)\;\}$$

Here, *r* is the pseudorandom number generated by the PRNG g() using a seed $s_{j}$. With the Butterfly algorithm, we facilitate the seeds to be inverted bitwise, beginning with the least-significant bit (LSB), until a chosen bit *j*. This means that the bits in the seed will be inverted (or, changed) one at a time beginning with the LSB until bit *j*. In other words, the Butterfly seed generation can be thought of as a function ϕ() that transforms a PRNG seed (*S*) into a variant of the same, as indicated by Equation (2).

(2) $$s_{j}=\phi \left(\;S\;\right)$$

Such design of a seed update mechanism allows flexibility in implementation, facilitating varying levels of unpredictability. This is because the implementation could take one of the following forms—(a) with each seed update, only one bit, identified by *j*, can be inverted, which would mean that the implementation would only need to offset to the particular bit and invert it, something that can be realized using shift registers (on hardware), applicable in severely resource-constrained applications; or, (b) with each seed update, all bits until and including the bit *j* can be inverted, implying that it would change the seed significantly, leading to higher unpredictability. An additional unpredictable element can be introduced in the choice of *j*. In simple implementations, *j* could be merely an incrementing number that identifies the position of the bit to be inverted. In an alternative implementation, it could be a number chosen at random that indicates the specific bit (or set of bits) to be changed.

Although this is not a direct replication of the Butterfly effect or the mathematical theory governing it, we see that our adaptation facilitates increasing the overall uncertainty in a system, thereby contributing to increasing security especially when algorithms used for PRNG and encryption are published. Furthermore, there would be no predictable pattern in the seed to an adversary (if he does not know the initial seed)—it would depend on the initial seed, and one bit of the seed would be changed at the end of each period of the PRNG (or on demand).

Our adaptation of the Butterfly effect, thus, facilitates design of key management and challenge-response authentication mechanisms, which have an implicit mechanism that increases the overall unpredictability, ultimately leading to increased security.

### 3.2. Encryption Scheme Employing Butterfly Seed Generation

In this section, we briefly discuss the encryption scheme and associated protocol that employs the Butterfly algorithm for seed update. This scheme was initially proposed for RFID systems, however, it can be extended to other resource-constrained systems such as WBANs, as we discuss in the next section.

**Figure 1:**
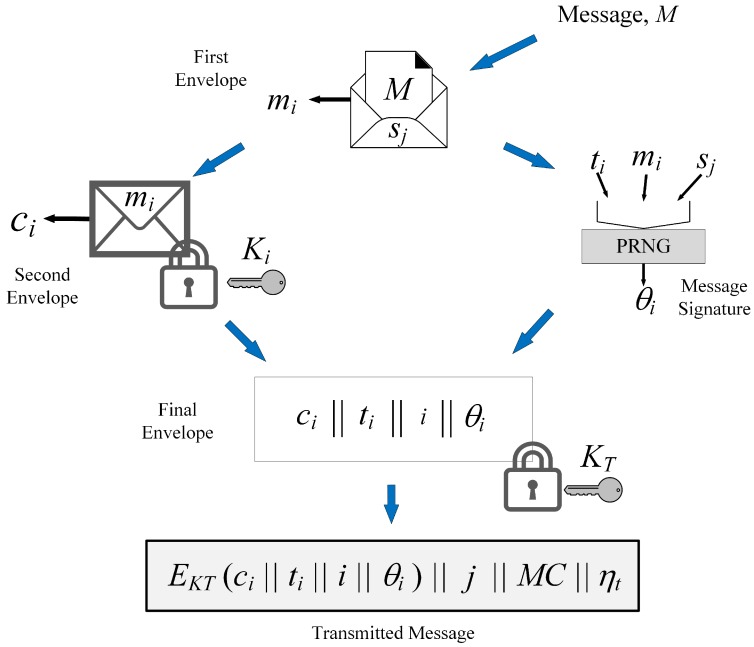
Working of the proposed encryption scheme [8].

Concepts central to the encryption scheme are enveloping and reuse. This scheme uses multiple envelopes to add to the security and reuse of functions (such as PRNG) to ensure reduced resource usage. This scheme uses the Butterfly seed update algorithm to generate unique keys for each encryption cycle. The scheme works as illustrated in Figure 1. For our discussion, we consider that the PRNG g() is initialized with seed $s_{init}$ and that the current seed is $s_{j}$. The message and the encryption cycle are identified by the sequence number *i*.

#### 3.2.1. Preliminaries

When required to transmit a message *M*, the entity first updates the seed to determine the updated seed $s_{j}$ using Equation (2). Following this, the entity encrypts (using XOR operation) the message using $s_{j}$ as the key (Equation (3)). This is the first envelope around the message to be transmitted.

(3) $$m_{i}=M⊕s_{j}$$

Further, let $t_{i}$ be the timestamp that the communication initiator retrieves prior to communication and $\theta_{i}$ be the message signature of $m_{i}$. Communicating entities store IDs and other information associated with the authenticated entities in memory (e.g., server storing ID and key information for an RFID tag in RFID systems). We assume that each such record associated with an entity in a database (or, a ‘row’ entry in a database) is identified uniquely by a number, $\eta_{t}$.

To accomplish the enveloping concept, two keys are used by this scheme (in addition to $s_{j}$ itself being used as a key, Equation (3)). These are the data encryption key $K_{i}$ and the transfer key $K_{T}$. Both keys are generated by PRNG as follows:

(4) $$K_{i}=g\left(\;f\left(\;\phi \left(\;s_{j}\;\right),\;t_{i}\;\right)\;\right)$$

(5) $$K_{T}=g\left(\;s_{j}\;\right)$$

Here, f() is the combination function, which combines input parameters. In our implementation, we used the XOR operation for combination. If and when required depending on the needs of the application, the combination function can be replaced without affecting the overall design of this scheme. $K_{i}$ is used to encrypt $m_{i}$ adding a second envelope over the data, while $K_{T}$ is used to encrypt the final message sent by the entity.

Data encryption can be accomplished by any symmetric encryption algorithm, such as AES [27], however, we used XOR to keep computations and resource usage low. The advantage of using XOR for encryption, especially when designs are implemented on hardware, is that XOR’s involutory nature makes it possible to use the same function for both encryption and decryption. (XOR is its own inverse. This is the involutory property of the XOR function.) Once again, although we used XOR in our description of this proposal, any symmetric encryption algorithm can be used with this scheme. Data encryption employed in this scheme is governed by Equation (6), where $cipher_{x}$ is the cipher text, $E_{Key_{x}}\left(\right)$ represents encryption of message $message_{x}$ with key $Key_{x}$, and ⊕ indicates the XOR function. Note that the parameters used in Equation (6) will change depending on the stage of encryption, *i.e.*, $Key_{x}=K_{i}$ when encrypting $m_{i}$ and $Key_{x}=K_{T}$ in the final encryption.

(6) $$cipher_{x}=E_{Key_{x}}\left(\;message_{x}\;\right)=message_{x}⊕Key_{x}$$

Another aspect we considered in this scheme was to use PRNG for generating message signatures. Although it is typical to use hash functions to generate message digests (used as signatures) [22], PRNG have the ability to generate unique sequences given a specific seed. We therefore used PRNG to generate message signature, $\theta_{i}$, for a given message and time (Equation (7)). Note that the combination function, f(), combines the current seed, message and timestamp to identify the context in the particular message transfer.

(7) $$\theta_{i}=g\left(\;f\left(\;s_{j},\;m_{i},\;t_{i}\;\right)\;\right)$$

#### 3.2.2. Protocol of Operation

**Figure 2:**
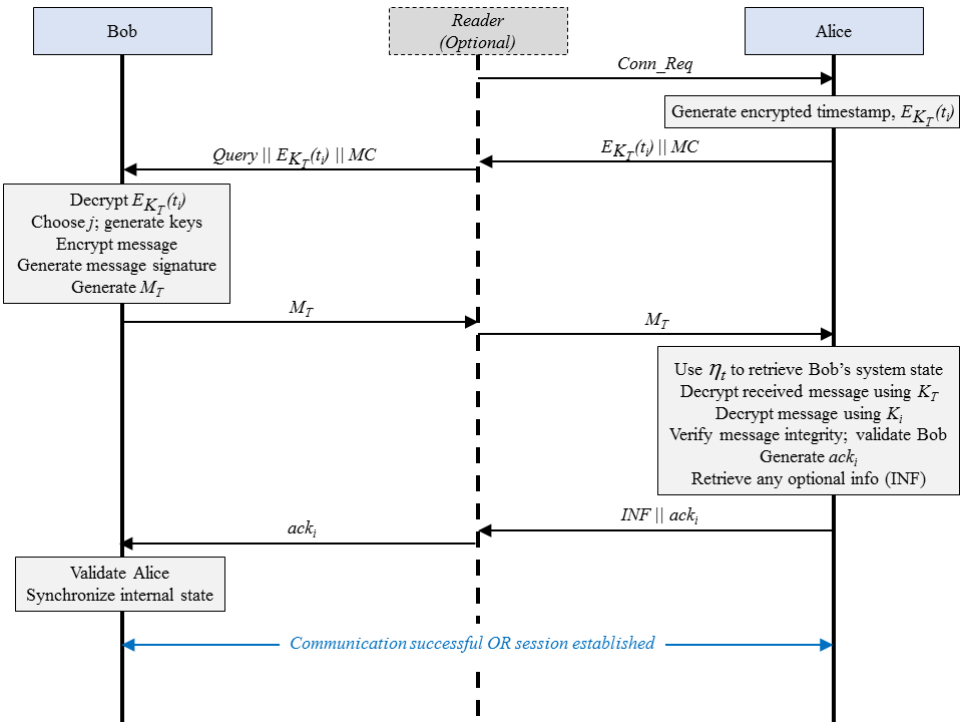
Protocol of Operation (Generalized Version of the Protocol Presented in [8]).

The protocol of operation is illustrated in Figure 2. (Note that the reader is shown as an optional entity to indicate that the use of intermediary devices in communication, as with using RFID readers in RFID systems, are optional and limited to specific resource-constrained networks.)

When communication begins, *i.e.*, either initiated by an intermediary through a communication request or by the entity, Alice, the first step involves the entity retrieving the current timestamp, $t_{i}$. Alice encrypts this using the synchronized transfer key $K_{T}$ and transfers $M0=E_{K_{T}}\left(\;t_{i}\;\right)∥MC$ (where ∥ represents concatenation). The parameter, MC, is the 2-bit message code, which indicates the type of message being communicated. The possible messages, identified by various message codes, are summarized in Table 1. In case of M0, MC is set to 0.

On reception of this message, Bob chooses a random integer value for *j* (0≤j≤m, with *m* being the length of the seed in bits). Bob retrieves the previously synchronized transfer key and extracts the timestamp $t_{i}$. Following this, Bob computes seed $s_{j}$ (Equation (2)) and generates keys $K_{i}$ and $K_{T}$. It computes $m_{i}$ (Equation (3)), and following this the cipher text $c_{i}$ (Equation (6)). It uses the various computed parameters to generate message signature, $\theta_{i}$ (Equation (7)). It then assembles its response, $M_{T}$ as follows:

(8) $$M_{T}=E_{K_{T}}\;\left(\;c_{i}∥t_{i}∥i∥\theta_{i}\;\right)∥j∥MC∥\eta_{t}$$

Note that $c_{i}$, $\theta_{i}$, $t_{i}$ and message sequence number, *i*, are encrypted using $K_{T}$ as the final stage of encryption or final envelope. This is indicated by $E_{K_{T}}\left(\right)$ in Equation (8).

**Table 1:** Message Code (MC) format and description [8].

MC	Bit Pattern	Description
MC=0	00	First message sent by Alice
MC=1	01	Response sent by Bob (Equation (8))
MC=2	10	Acknowledgement sent by Alice (Equation (10))
MC=3	11	Special message: Instructs Bob to update
		its PRNG seed, if Alice is valid (Equation (11))

#### 3.2.3. Decryption

On receiving $M_{T}$, Alice uses *j* to compute $s_{j}$, and hence, $K_{i}$(using the stored timestamp, $t_{i}$) and $K_{T}$. It uses $K_{T}$ to retrieve $c_{i}$, $\theta_{i}$, $t_{i}$ and message sequence number, *i*. Next, it computes $\theta_{i}$ to verify message integrity. Bob’s authenticity is verified if $\theta_{i,received}$ is equal to $\theta_{i,generated}$. This is because only an authorized entity with synchronized states will be able to generate the proper keys using $s_{j}$. Following its verification, Alice recovers $m_{i}$ and hence message *M*. Following this, Alice transfers an acknowledgement pattern ($ack_{i}$) with the new timestamp ($t_{i+1}$) and transfers this with any optional messages to be transferred to the intermediaries (Equations (9) and (10)).

(9) $$c_{ack}=E_{K_{i}}\;\left(\;ACK∥t_{i+1}\;\right)$$

(10) $$ack_{i}=E_{K_{T}}\;\left(\;c_{ack}∥i∥\theta_{i}\;\right)∥MC$$

Here, $c_{ack}$ is the acknowledgement cipher, $K_{T}$ and $K_{i}$ are the same keys used by Bob, ACK is the pre-agreed acknowledgement pattern, $t_{i+1}$ is the latest timestamp at Alice, *i* is the previous sequence number sent by Bob, and MC=2 to indicate acknowledgement. Values of $K_{T}$, $K_{i}$, *i* used here are those used by Bob and this helps in synchronization.

#### 3.2.4. Seed Refresh

In case either entity wants to update or refresh the seed, they can send a seed refresh message, $m_{su}$, as specified by Equation (11). The message $m_{su}$ contains the seed update cipher ($c_{u}$), which the seed update initiator computes. This contains the combination of the current seed, $s_{j}$ and the new seed, $s^{\prime }$, encrypted using $K_{i}$ (as per Equation (4)). Furthermore, in $m_{su}$, $t_{u}$ is the current timestamp and the generated message signature is $\theta_{u}$. In this case, *j* is the Butterfly state randomly chosen by the initiator of the seed update and MC=3.

(11) $$m_{su}=E_{K_{T}}\;\left(\;c_{u}∥t_{u}∥i∥\theta_{u}\;\right)∥j∥MC∥\eta_{t}$$

**Algorithm 1:** Key Generation and Encryption in the Butterfly Encryption Scheme**Input:** Seed bit identifier (*j*), updated seed ($s_{j}$), timestamp ($t_{i}$). **Output:** Transmitted message ($M_{T}$) or Seed update response ($m_{su}$). Choose *j*;
$s_{j}\leftarrow \phi \left(\;s\;\right)$;
$K_{i}\leftarrow g\left(\;f\left(\;s_{j},t_{i}\;\right)\;\right)$;
$K_{T}\leftarrow g\left(s_{j}\right)$; //Choose appropriate message code (2 bits):
**if** (*First message,*
Bob←Alice) **then**
MC←00;
**else if** (*Response,*
Alice←Bob) **then**
MC←01;
**else if** (*ACK,*
Bob←Alice) **then**
MC←10;
**else if** (*Special message*) **then**
MC←11; $m_{i}\leftarrow M\;⊕\;s_{j}$; $c_{i}\leftarrow m_{i}\;⊕\;K_{i}$; $\theta_{i}\leftarrow f\left(\;t_{i}∥m_{i}∥s_{j}\;\right)$; 
**if**
MC=00
*or*
MC=01
*or*
MC=10
**then**
$E_{K_{T}}\left(params\right)\leftarrow \left(\;c_{i}∥t_{i}∥i∥\theta_{i}\;\right)\;⊕\;K_{T}$;//Generate Message to be Transmitted:$M_{T}\leftarrow E_{K_{T}}\left(params\right)∥j∥MC∥\eta_{t}$;
**else if**
MC=11
**then**
$c_{u}\leftarrow c_{i}$, $\;\;t_{u}\leftarrow t_{i}$, $\;\;\theta_{u}\leftarrow \theta_{i}$;//Generate Seed Update Message:$m_{su}=E_{K_{T}}\;\left(c_{u}∥t_{u}∥i∥\theta_{u}\right)∥j∥MC∥\eta_{t}$;
**end**

The pseudocode for the Butterfly encryption scheme is summarized in Algorithm 1. This encryption scheme was proposed for RFID systems, to ensure security due to high unpredictability introduced by the changing seed states and random choices. In the next section, we discuss a use case of extending this scheme to be applied to Wireless Body Area Network (WBAN) applications.

## 4. Extending Butterfly Encryption Scheme to WBANs

RFID tags, especially Class-0/1 passive tags [28], are even more resource-constrained than WBAN on-body sensors. This is primarily because of the absence of an on-chip power source, which limits the operating time of the tag. The Butterfly encryption scheme was proposed for RFID systems as a lightweight security proposal.

**Figure 3:**
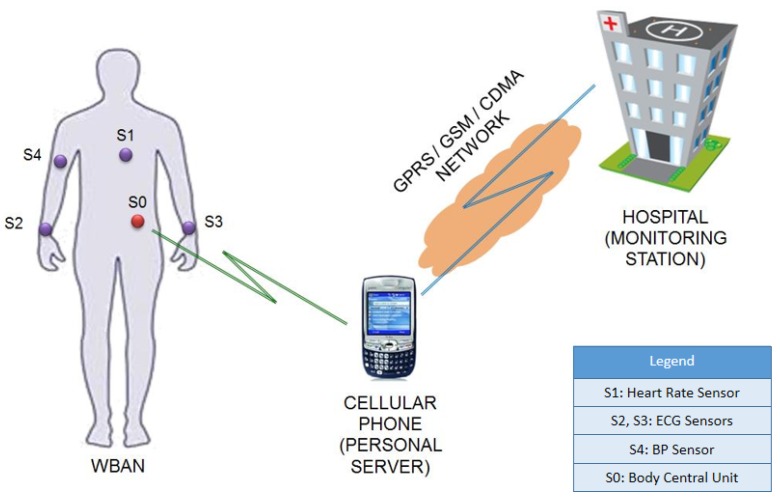
A Typical WBAN System [29].

In WBANs, a sparse network of sensors (“Body Sensor Units”, BSUs) are deployed either directly on the human body, inside the body or embedded in everyday clothes, to record and transmit health data. The BSUs record and transmit data to a Body Central Unit (BCU) or the “WBAN hub” [30], which aggregates data sent by all BSUs and relays the aggregation to a hospital monitoring station. However, the hub might not have sufficient infrastructure to relay the data directly to a monitoring station, and therefore typically uses a Personal Server (PS) as an intermediary. A personal server can be either fixed or mobile, typical examples of which include cellular phones, personal digital assistants (PDAs), desktop or laptop computers [3]. A typical setup of wireless body area networks is illustrated in Figure 3, with BSUs—S1 (heart rate, measured in beats per minute, bpm), S2 (blood pressure in millimeters of mercury, mmHg), and S3 (blood glucose level in milligrams per decilitre, mg/dl), and S0 being the BCU, using cellular phone as a personal server.

WBAN sensors are connected to a power source (batteries), which enables them to perform the required “sensing” operation, among other tasks. However, the presence of a power source does not imply that the sensors can be always on, since that would increase the battery consumption and require frequent maintenance (battery replacement). For optimal performance, sensors may be programmed to enter “sleep” state following message transmission, for a predetermined duration of time [4]. This, in a way, makes them similar to active RFID tags.

Our approach can be used to protect the communication between the sensor nodes and the WBAN body central node or the hub (*phase 1*), and can also be employed for the communication between the hub and the personal server (*phase 2*). With the personal server and the monitoring station known to have higher computational abilities, we do not consider their security in the purview of our work. Butterfly can be employed in both phase 1 and phase 2 communication in WBANs. When we consider phase 1, we assume that each sensor node has a specific identifier (ID) that the protocol identifies as $\eta_{t}$ and that each node has a specific seed $s_{n}$ that is stored at deploy time. The hub stores all these seeds and IDs in memory, and maintains a separate seed for its own communication with the personal server. Note that the reader role is eliminated in this application of the encryption scheme.

Our approach also facilitates either entity to be able to demand the other generate specific parameters for encryption and authentication. This begins with the random choice of *j* at the initiator and retrieval of the timestamp ($t_{i}$). If the initiator is the sensor node, it computes the keys and the message signature based on the value of *j* and $t_{i}$, and transmits the encrypted recorded data to the hub as specified in Section 3.2. Following this, the hub authenticates the node using $\eta_{t}$ and *j*, and sends an acknowledgement on successful authentication. The same mechanism can be applied when used in phase 2 of the WBAN communication, with the personal server and the hub generating specific parameters on demand, as decided by *j* and $t_{i}$.

The simplicity of the Butterfly encryption scheme enables us to extend it to other resource-constrained application domains, such as WBANs, with negligible changes or adaptations. If and when the scenario demands, the Butterfly algorithm can also be extended to generate longer keys (e.g., 256 bits, 512 bits, or more), which would also add to the security. This however will be dependent on the symmetric encryption algorithm that is used to encrypt messages. We used XOR in our implementation, however, any other symmetric encryption algorithm can be used.

Our proposal is primarily focused on key generation and authentication through message signatures, whether it is considered in its initial form proposed for RFID systems [8] or as extended to WBANs. The foundation for this is the Butterfly seed update algorithm, based on a naïve adaptation of the Butterfly effect. This is an attempt to reuse available functions to accomplish multiple functions such as key generation, encryption, message signature generation, which results in achieving security objectives such as confidentiality, integrity, (mutual) authentication, and non-repudiation (by association). Simplicity and reuse in this approach encourage its application in other resource-constrained wireless applications such as WBANs, as discussed in this section. In the next section, we discuss the evaluation of our encryption scheme, assessing the encryption keys generated and the overall security of the system. We also present a discussion on the resource utilization by this scheme.

## 5. Evaluation and Results

Our evaluation of the Butterfly encryption scheme involved three parts:

- *Key Sequence Evaluation*: We implemented our approach using Java programming language to verify the working of the concept and generation of key sequences for further evaluation. We used the generated key sequences to test similarity between consecutive keys using Sörensen’s Similarity Index (SSI) [31] and to verify the randomness in key sequences using the Statistical Test Suite (STS) published by NIST (National Institute of Standards and Technology) [32]. Using the results so obtained, we compared the performance of our approach with the RFID security proposals by Zhu *et al.* [13] and Dong *et al.* [12], and with an AES-based key generation approach for WBANs proposed by Liu *et al.* [20]. Note that we also implemented the proposals by Zhu *et al.*, Dong *et al.*, and Liu *et al.* using Java to generate key sequences for our assessment.
- *Hardware Complexity Evaluation*: We estimated the approximate resource requirement for implementing the Butterfly encryption scheme on hardware.
- *Security Evaluation*: We also performed a security assessment using Scyther protocol analyzer [33,34,35,36] and qualitative security analysis to evaluate the security of our encryption scheme.

### 5.1. Key Sequence Evaluation

#### 5.1.1. Methodology

We implemented the Butterfly seed update algorithm and the encryption scheme using Java to first verify the concept. In our implementation, the PRNG used to choose the value of the variable *j* at random, which decides the state of the seed ($s_{j}$), was initialized to 192BC333250CCCFF, while the seed (*s*) itself was initially set to 12345678. We used the Java Random class to introduce random delays (0 and 2 s) between consecutive key generations, as an attempt to emulate real-time communication, and used methods in the Random class to extract PRNG sequences. Furthermore, we used the Java method System.currentTimeMillis() to extract the timestamp. We extracted 10240 key sequences for our assessment.

According to Kerckhoff’s principle [5], the knowledge of the operational specifics of a cryptosystem (except the key) must not reduce its security. This led us to assess the keys generated by our approach using two aspects—similarity between consecutive keys and randomness/unpredictability in the individual sequences. To extract the characters in each key and quantify similarity between consecutive keys, we used SSI, which is computed as summarized by Equation (12).

(12) $$SSI=\frac{2\times n(A\cap B)}{n(A)\;+\;n(B)}$$

Here, n(A∩B) represents the number of characters (or, numbers) in the key pair that are the same, n(A) and n(B) represent the total number of characters (or, numbers) in each of the keys A and B of the key pair, respectively.

Following the assessment of similarity between key sequences, we evaluated the randomness in each key sequence using NIST STS [32]. STS is primarily used to assess the randomness and unpredictability of sequences generated by random and pseudorandom number generators. Although our proposal is not intended as a random or pseudorandom number generator, we used it to assess the key sequences since it serves as a means to evaluate the randomness associated with the generated keys. Randomness is a measure of security, since the more random sequences are, the harder it will be for an adversary to crack or guess. This helps us assess the unpredictability associated with our scheme, and hence security. We used 10000 (128-bit) key sequences for this assessment (sample size, *m*). STS determined the range of acceptable proportions, *i.e.*, the confidence interval, using Equation (13) [32].

(13) $$Confidence\;interval,CI=\hat{p}\pm 3\sqrt{\frac{\hat{p}\left(1-\hat{p}\right)}{m}}$$

Here, $\hat{p}$ is (1-α), with *α* being the significance level. In our assessment, α=0.01 and sample size m=10000. The confidence interval is $CI=0.99\pm 3\sqrt{\frac{0.99(1-0.99)}{10000}}=0.99\pm 0.00298496$. Therefore, for a set of sequences to be considered random, the minimum (acceptable) number of sequences that have to pass the test are (0.99-0.00298496)×10000=0.98701504×10000≈9870 sequences. To evaluate the randomness, we considered the following tests in STS—Frequency (monobit) test, Frequency tests within a block (block size = 32 bits), Runs test, Longest runs of ones in a block, and Discrete Fourier transform (DFT) test.

#### 5.1.2. Results

**Figure 4:**
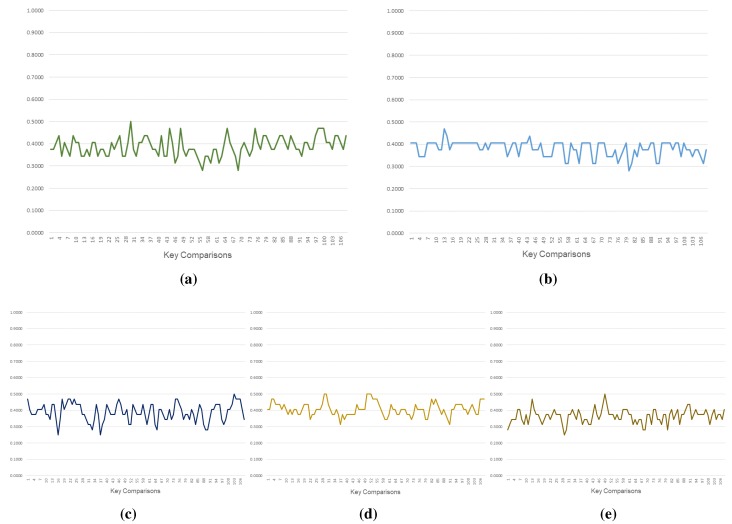
Variation in SSI coefficients. (**a**) Butterfly ($K_{i}$); (**b**) Butterfly ($K_{T}$); (**c**) Liu *et al.*’s proposal; (**d**) Zhu *et al.*’s proposal; (**e**) Dong *et al.*’s proposal.

Figure 4 illustrates the variation for the first 108 key comparisons (note that we have illustrated only 108 key sequences to demonstrate the variation within the space confines of a page). Table 2 summarizes the average SSI values over 10239 key comparisons. $K_{i}$ displays considerable variation, which can be attributed to the changing values of timestamp and $s_{j}$, that generate these keys. We also observe that the variation in $K_{T}$ is lower as compared to $K_{i}$. This can be attributed to the random choice of the numerical value of *j*, which when chosen at random could result in the same value, and hence the same bit being inverted. In our implementation, we considered the option of inverting only one bit identified by *j* (option (a) discussed in Section 3.1). Although the transfer key remains the same for a few communications in our proof-of-concept implementation, we must note that the value of $K_{i}$ keeps changing, which implies that the cipher resulting from encrypting the parameters using $K_{T}$ also keeps changing, minimizing the effect of reduced variation. This serves as a way of increasing the uncertainty associated with a transmitted message, as the contextual parameters that change with each message ensure that the communication remains secure.

In comparison with other key generation schemes [12,13,20], we observe that the keys generated by our approach perform better in terms of similarity (or, ensuring dissimilarity). The keys generated in the proposal by Zhu *et al.* [13] includes a scheme of updating the keys block-wise, which retains the other (non-updated) blocks of keys similar to the previous keys, increasing the average SSI.

**Table 2:** Summary of similarity between keys.

Configuration	Average SSI ($SSI_{av}$)
Butterfly ($K_{i}$)	0.3809
Butterfly ($K_{T}$)	0.3833
Liu *et al.* [20]	0.3826
Zhu *et al.* [13]	0.4110
Dong *et al.* [12]	0.3815

Evaluation of similarity between keys helped us determine that consecutive keys are dissimilar, which was an attempt to verify whether similarity could be exploited by adversaries to be able to crack or guess keys. To continue this attempt at evaluating the unpredictability of keys, we assessed the randomness in individual key sequences. STS demonstrated that all sequences considered for assessment passed the criterion (>9870). With regard to the Discrete Fourier Transform test, however, our results indicated that although the variability in all schemes considered is sufficiently high, there are a few sequences in which the periodic features are found to be higher than would be expected in a random sequence. Based on the STS results in Table 3, we also observed that the sequences generated by our Butterfly approach perform at par to sequences generated using standard algorithms such as AES (Liu *et al.* [20]), SHA-3 (Dong *et al.* [12]), and the block-wise key update mechanism by XTEA (Zhu *et al.* [13]).

The passed sequences indicate that the key sequences generated by our scheme are able to pass the tests for randomness, when assessed with the expectations for a random or a pseudorandom number generator. This is an encouraging aspect, since it highlights the randomness associated with individual key sequences, thereby supporting our claims of increased unpredictability, and thus security.

**Table 3:** NIST STS Assessment: Summary of results (Number of sequences out of 10,000 that passed each test).

Configuration (Keys Considered)	FM Test ${}^{1}$	FB Test ${}^{2}$	Runs Test	LR Test ${}^{3}$	DFT Test ${}^{4}$
Butterfly ($K_{i}$)	9895	9908	9906	9896	9862
Butterfly ($K_{T}$)	9906	9906	10,000	9917	10,000
Liu *et al.* [20]	9913	9914	9888	9912	9837
Zhu *et al.* [13]	9891	9892	9892	9886	9832
Dong *et al.* [12]	9898	9926	9890	9894	9833

${}^{1}$: Frequency (Monobit) Test; ${}^{2}$: Frequency Test within a Block; ${}^{3}$: Longest Runs of Ones in a Block; ${}^{4}$: Discrete Fourier Transform (Spectral Test).

### 5.2. Hardware Complexity Evaluation

To estimate the approximate resource requirement for implementing our approach, we estimated the logical circuit blocks that might be necessary for a hardware implementation of our scheme, considering that some of the blocks could be reused for different functions. If the size of the message (following operations such as concatenation) increases to beyond maximum length of the message supported by such reused functions (especially in hardware implementations), the data to be processed is processed as blocks of maximum allowed length. For example, if an *n*-bit XOR circuit is used, then, data will be processed as blocks of *n* in case the final data to be processed has a length l>n. We summarize the logic circuit estimation in Table 4 (note that *n* is the size of the key in bits).

**Table 4:** Logic Circuit Estimation.

Logic Circuit	Count
PRNG	*n* bit ×1
Addition	$\frac{n}{2}$ bit ×1
XOR *	*n* bit ×1
NOT (Invert)	1 bit ×1
MUX (Multiplexer)	2:1×1

* XOR is used for encryption and as combination function in our scheme.

### 5.3. Security Evaluation

We performed a security assessment of the protocol of operation (Figure 2) using Scyther protocol analyzer [33,34,35,36]. Scyther uses the Dolev and Yao adversary model [33,37], which assumes perfect cryptography, abstract messages and that the adversary has full control over the network. In the paragraphs that follow, we discuss the security claims [35,36], the associated results and their implications on some network attacks, while we summarize the Scyther assessment results in Table 5. In our discussion of the Scyther results, we also include how each claim verified by Scyther impacts the behavior of our scheme with respect to standard security goals [38]—confidentiality, integrity, authentication, non-repudiation (by association) and forward/backward secrecy.

**Table 5:** Results: Evaluation of the Protocol Using Scyther [8].

Claim	Bob		Alice	
	**Status**	**Comments**	**Status**	**Comments**
Secret $m_{i}$	OK	NAWB *	OK	NAWB
Secret $t_{i}$	OK	NAWB	OK	NAWB
Secret *i*	OK	NAWB	OK	NAWB
Secret i1	OK	NAWB	OK	NAWB
Secret *θ*	OK	NAWB	OK	NAWB
Secret $s_{j}$	OK	NAWB	OK	NAWB
Secret $K_{i}$	OK	NAWB	OK	NAWB
Secret $K_{T}$	OK	NAWB	OK	NAWB
Secret $s_{j,new}$	OK	NAWB	OK	NAWB
Secret $t_{i,new}$	OK	NAWB	OK	NAWB
Secret θ1	OK	NAWB	OK	NAWB
Alive	OK	NA-Verified ${}^{†}$	OK	NAWB
Weakagree	OK	NA-Verified	OK	NAWB
Nisynch	OK	NAWB	OK	NAWB
Niagree	OK	NAWB	OK	NAWB

* NAWB = No attacks, within bounds; ^†^ NA-Verified = No attacks, verified.

*Claim-1: Secrecy*: The following parameters were expected to be secret in the communication between entities: $s_{j}$, $K_{i}$, $K_{T}$$m_{i}$, $t_{i}$, *i*, $\theta_{i}$, $t_{u}$, $\theta_{u}$, $c_{u}$).

*Result*: With keys dynamically updated using timestamps, the Butterfly protocol of operation ensures that all secrecy claims are valid. This implies that the secrecy of the message $m_{i}$ and the updated seed $s^{\prime }$ are both maintained a secret, thereby helping to achieve *confidentiality*. It has to be noted that even the message signatures $\theta_{i}$ and $\theta_{u}$ are maintained a secret, since these are included in the second envelope encrypted with $K_{T}$, generated using seed $s_{j}$. This implies that the *integrity* of the message can be verified.

*Claims-2 and 3: Alive and Weakagree*: Previous messages have been encrypted using the same scheme, and entities are using the same scheme in their communication.

*Result*: The protocol analyzer validates our claims that the entities are running the same scheme (Weakagree) and all previous message sessions have used the proposed scheme (Alive).

*Claim-4: Non-injective Synchronization (Nisynch)*: Prior communication sessions between entities have been governed by the specifications of the protocol and entities are synchronized to their current state.

*Result*: The analyzer validates our claim that the protocol and the scheme ensure that the internal key generation states are synchronized in the communicating entities. This is possible due to the presence of timestamps, sequence numbers and message codes, which not only protect the system against *replay attacks*, but also render *de-synchronization attacks* ineffective. This is augmented by the multi-level enveloping technique employed by our scheme.

*Claim-5: Non-injective Agreement (Niagree)*: The communicating entities agree upon some parameters during the course of a session.

*Result*: The first seed $s_{init}$ is a central attribute in our scheme. This is never shared openly or even through encrypted messages. Its variant $s_{j}$ is used for generating multiple keys, and a new seed $s^{\prime }$ is also communicated in an encrypted manner. This dependency on the initial seed protects the scheme operation since it makes it harder for an adversary to guess the particular (current) state of a seed. This is an important pre-agreed parameter, and the analyzer validates our claim that the entities can agree upon important parameters (such as $s^{\prime }$) during communication using this protocol.

*Authentication and Non-repudiation*: The receiver can authenticate the initiator from the transmitted message, $M_{T}$, using *η*, pre-shared secret seed $s_{j}$ and the value of *j* to generate encryption keys to decrypt the received data. By computing $\theta_{i}$, it is able to confirm the identity of the sender. Furthermore, the initiator can validate the responder upon receipt of the acknowledgement message, when it decrypts $ack_{i}$ to generate $\theta_{i}$ that it had previously sent. Thus, both entities can authenticate each other. Since the initial seed is pre-shared and never exchanged, only a legitimate entity will be able to generate specific values of $\theta_{i}$ and the keys $K_{i}$ and $K_{T}$.

This implies that for each entity to be in continuous synchronization, neither of them can deny the messages they send. This, in a naïve manner, helps in accomplishing *non-repudiation*. Although symmetric cryptosystems cannot guarantee non-repudiation without the use of asymmetric components/digital signatures [37], our approach facilitates non-repudiation by association. (Non-repudiation by association implies accomplishing this goal by being associated with a backend server, which can be authenticated by another trusted entity using trusted third parties and digital signatures.) Since entities are communicating with a server with unlimited resources, we assume the server to be authentic, and by verifying itself to the server, the resource-constrained entity guarantees that the messages sent by it are valid.

*Forward and Backward secrecy*: The use of timestamp to generate the encryption key $K_{i}$ continuously changes the seed $s_{j}$ and hence the transfer key $K_{T}$, which helps in ensuring secrecy of data. Even if a contiguous set of keys were to be determined by an adversary, it would be challenging to determine either past keys or future keys, since the internal PRNG seed is not disclosed and the computed keys are dependent on the changing parameters. Thus, our approach helps preserve forward and backward secrecy, adding to the overall security of the system.

## 6. Discussion

Our approach is primarily proposed as a key generation and authentication mechanism for resource-constrained applications. Using simple operations such as pseudorandom number generation and XOR, our scheme accomplishes several security objectives without requiring extensive resources. The strength of this approach is in its ability to use pre-shared parameters with minimal changes in each message transmission, which allows generation of fresh encryption keys for each message. This introduces an element of uncertainty to the cryptosystem, which makes it harder for unauthorized entities to ‘guess’ the keys of the system. This improves security. Furthermore, by employing a multiple enveloping mechanism, or including multiple rounds of encryption, our scheme ensures that there is an implicit complexity that improves security.

The results presented in Section 5 substantiate our claim of the key sequences being sufficiently random and the reduced similarity between consecutive keys. These results, in addition to reduced resource requirements, show that our scheme can indeed be deployed in resource-constrained devices, and accomplish standard security objectives. Security of the messages encrypted using a scheme is also dependent on its ability to ensure that its parameters are secret, and that the entities are able to successfully communicate using said scheme. Our assessment of the protocol of operation using Scyther supports the ability of the scheme to accomplish secrecy over communication, while ensuring synchronized states. With keys being generated and each key determined using a secret seed, whose state is chosen at random, the reliance on a resourceful entity (such as a server) to compute keys for a resource-constrained entity is eliminated. The implicit challenge-response mechanism of mutual authentication also guarantees that entities are able to not only generate encryption parameters but also authenticate each other, while ensuring synchronized states.

Perhaps the biggest benefit of this scheme is its ability to be extended to any domain, beyond resource-constrained applications. The use of timestamps enables it to be deployed in scenarios that might necessitate varying keys in the duration of a session. Our scheme facilitates use of continuously changing keys within a session without complex computation, which means that the overall uncertainty and hence, security of such a system can be increased.

Controlled changes to the seed using the Butterfly seed update algorithm helps ensure that the PRNG seed changes with each message, reducing the probability of any unauthorized entities determining the key sequences generated as a result. Changing seeds and timestamps ensure that there is an element of context to the communication, making it meaningful only to authorized entities.

## 7. Conclusions

In this paper, we presented an approach to update seeds and generate keys at communicating entities. Continuously updating PRNG seeds and using timestamps enable entities to be able to generate keys and authentication parameters to accompany encrypted messages. Multi-level encryption employed by our scheme ensures that the actual data is substantially cloaked. Our approach features a naïve adaptation of the Butterfly effect to update seeds, a mechanism that helps in accomplishing mutual authentication as well. The simplicity in design enables our scheme to be deployed in resource-constrained entities, where security is desired but increased computation is too costly and thus unwanted.

The use of changing parameters and timestamp ensures that there is sufficient unpredictability associated with the system, making it harder for unauthorized entities to decipher. Our analyses substantiate our claim of low resource requirement and high unpredictability. Furthermore, our scheme facilitates entities to be able to sign their own messages and accomplish non-repudiation by association.

Depending on the needs of the application, our scheme can be deployed with any symmetric cryptosystem to generate keys and authentication parameters. This would depend on the available resources and the security requirements, in addition to factors such as cost and required longevity of the devices.

We set out to design a simple scheme that is able to accomplish unpredictability and security through key generation (rather than exchange), generation of message signatures, and that employs an element of randomness (timestamp). Our Butterfly encryption scheme is able to accomplish all these objectives, while being suitable for resource-constrained devices, and facilitating its application in other domains as well.
